# Application of Mice Humanized for CYP2D6 to the Study of Tamoxifen Metabolism and Drug–Drug Interaction with Antidepressants[Fn FN3]

**DOI:** 10.1124/dmd.116.073437

**Published:** 2017-01

**Authors:** A. Kenneth MacLeod, Lesley A. McLaughlin, Colin J. Henderson, C. Roland Wolf

**Affiliations:** Division of Cancer Research, Level 9, Jacqui Wood Cancer Centre, School of Medicine, University of Dundee, Dundee, DD1 9SY, United Kingdom

## Abstract

Tamoxifen is an estrogen receptor antagonist used in the treatment of breast cancer. It is a prodrug that is converted by several cytochrome P450 enzymes to a primary metabolite, *N*-desmethyltamoxifen (NDT), which is then further modified by CYP2D6 to a pharmacologically potent secondary metabolite, 4-hydroxy-*N*-desmethyltamoxifen (endoxifen). Antidepressants (ADs), which are often coprescribed to patients receiving tamoxifen, are also metabolized by CYP2D6 and evidence suggests that a drug–drug interaction between these agents adversely affects the outcome of tamoxifen therapy by inhibiting endoxifen formation. We evaluated this potentially important drug–drug interaction in vivo in mice humanized for CYP2D6 (hCYP2D6). The rate of conversion of NDT to endoxifen by hCYP2D6 mouse liver microsomes (MLMs) in vitro was similar to that of the most active members of a panel of 13 individual human liver microsomes. Coincubation with quinidine, a CYP2D6 inhibitor, ablated endoxifen generation by hCYP2D6 MLMs. The NDT-hydroxylation activity of wild-type MLMs was 7.4 times higher than that of hCYP2D6, whereas MLMs from *Cyp2d* knockout animals were inactive. Hydroxylation of NDT correlated with that of bufuralol, a CYP2D6 probe substrate, in the human liver microsome panel. In vitro, ADs of the selective serotonin reuptake inhibitor class were, by an order of magnitude, more potent inhibitors of NDT hydroxylation by hCYP2D6 MLMs than were compounds of the tricyclic class. At a clinically relevant dose, paroxetine pretreatment inhibited the generation of endoxifen from NDT in hCYP2D6 mice in vivo. These data demonstrate the potential of ADs to affect endoxifen generation and, thereby, the outcome of tamoxifen therapy.

## Introduction

**Fig. 1.**
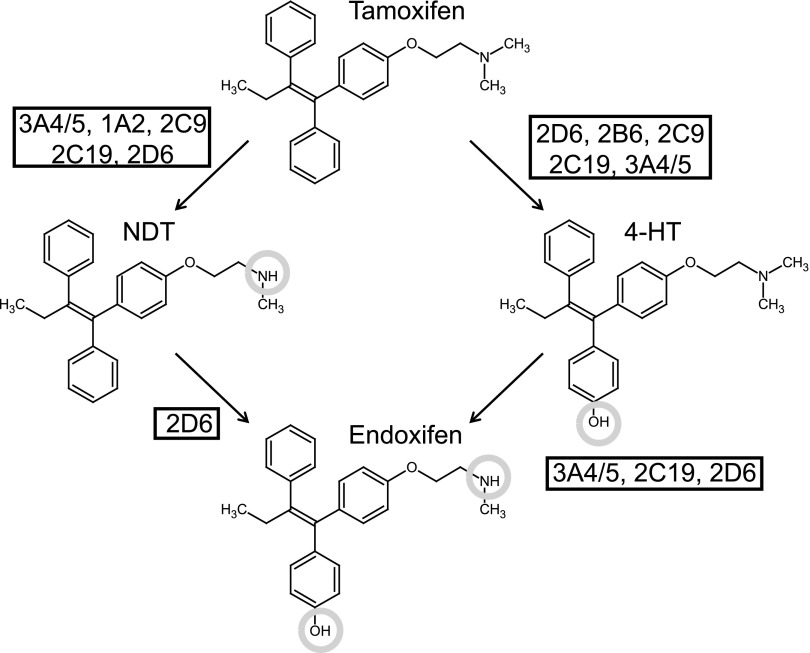
Phase I metabolism of tamoxifen. P450s metabolize tamoxifen through the “major” (*N*-demethylation followed by 4-hydroxylation) or “minor” (4-hydroxylation followed by *N*-demethylation) pathway, so denoted due to the relative abundance of each metabolite in plasma samples (Stearns et al., 2003; Madlensky et al., 2011). Conversion of NDT to endoxifen is catalyzed exclusively by CYP2D6 (Desta et al., 2004).

Although tamoxifen has been approved for clinical use for over 40 years, only recently has it been identified as a potential prodrug. Two hydroxylated metabolites in particular, endoxifen and 4-hydroxytamoxifen (4-HT), have been shown to be up to 100 times more potent estrogen receptor (ER) antagonists than the parent compound (Johnson et al., 2004) and are therefore likely to contribute to target inhibition and, thereby, the outcome of therapy (Fig. 1). Because endoxifen is several times more abundant in systemic blood samples than 4-HT, it is generally considered the more important of these metabolites (Stearns et al., 2003; Madlensky et al., 2011). Crucially, the rate-limiting step in the conversion of tamoxifen to endoxifen is catalyzed by the highly polymorphic enzyme, CYP2D6 (Desta et al., 2004). Phenotypic status with regard to this enzyme profoundly influences the circulating level of endoxifen at steady state (Stearns et al., 2003; Mürdter et al., 2011). The clinical significance of these observations has been scrutinized intensely, with large numbers of retrospective studies finding both for and against an effect on therapeutic outcome. Perhaps most notably, a meta-analysis by the International Tamoxifen Pharmacogenomics Consortium found, when strict inclusion criteria were applied, a clear association of CYP2D6 poor-metabolizer status with lower rates of invasive disease-free survival on tamoxifen therapy (Province et al., 2014).

In addition to the pharmacogenetic variability in CYP2D6 activity, this enzyme is also the focal point for a number of clinically significant drug–drug interactions. Between 10% and 25% of women with breast cancer experience depression (Fann et al., 2008), whereas as many as 70%–80% experience hot flashes (hot flushes; Day et al., 1999). In both instances, antidepressants (ADs) may be prescribed, many of which are both substrates for and inhibitors of CYP2D6. Available clinical data indicate a 45%–58% decrease in plasma levels of endoxifen in individuals taking CYP2D6 inhibitors (Stearns et al., 2003; Jin et al., 2005; Borges et al., 2006). Two of the most commonly used ADs, the selective serotonin reuptake inhibitors (SSRIs) paroxetine and fluoxetine, are classed as strong inhibitors of CYP2D6 (http://www.fda.gov/Drugs/DevelopmentApprovalProcess/DevelopmentResources/DrugInteractionsLabeling/ucm093664.htm#cypEnzymes) and these have an even greater bearing on the amount of circulating endoxifen, particularly in individuals carrying allelic variants of CYP2D6 that confer the extensive metabolizer phenotype, with decreases of 64%–71% (Stearns et al., 2003; Borges et al., 2006). In a retrospective population-based cohort study of 2430 individuals who received at least one antidepressant during tamoxifen therapy, paroxetine was found to be the only comedication associated with an increased risk of death from breast cancer (Kelly et al., 2010); however, this remains a contentious finding, because other studies have yielded conflicting results (Lehmann et al., 2004; Chubak et al., 2008; Ahern et al., 2009; Dezentjé et al., 2010; Lash et al., 2010).

Here, our aim was to use a CYP2D6-humanized (hCYP2D6) mouse model to study tamoxifen metabolism, with a particular focus on whether an in vivo interaction of tamoxifen and its metabolites with ADs could be demonstrated.

## Materials and Methods

### 

#### Chemicals and Reagents.

High/low cytochrome P450 (P450) activity human liver microsome (HLM) preparations from individual donors were purchased from BD Gentest (San Jose, CA). Pooled HLMs (150 donors) were purchased from Thermo Fisher Scientific (Waltham, MA). Endoxifen and tamoxifen-*d*_5_ were obtained from Toronto Research Chemicals (Toronto, ON, Canada). NADPH was purchased from Melford Laboratories (Ipswich, UK). All other chemicals were purchased from Sigma-Aldrich (Poole, UK).

#### Animal Lines and Husbandry.

The generation and characterization of Cyp2d-knockout (Cyp2dKO) and hCYP2D6 mice was described previously (Scheer et al., 2012). Briefly, all nine functional murine *Cyp2d* genes were deleted to produce the Cyp2dKO line, and the hCYP2D6 line was generated by a targeted insertion of an expression cassette containing 9 kb of the CYP2D6 promoter, along with all exons, introns, and 5′ and 3′ untranslated regions, into the murine *Cyp2d* locus. These animals were obtained from Taconic (Cologne, Germany) and were maintained by regular outcrossing to C57/BL6N, and they were backcrossed on the same genetic background for at least six generations. C57BL/6N mice were used as wild-type controls. Mice were housed on sawdust in solid-bottom, polypropylene cages and were provided an RM1 pelleted diet (Special Diet Services Ltd., Essex, UK) and drinking water ad libitum before and throughout the studies. The temperature was maintained within the range of 19–23°C, and the relative humidity was within the range of 40%–70%. A 12-hour light/dark cycle was maintained. All animal procedures were carried out on 8- to 12-week old female mice under the auspices of the Animal (Scientific Procedures) Act of 1986, as amended by European Union Directive 2010/63/EU, and after local ethical review.

#### Subcellular Fractionation.

Livers were excised and snap-frozen in liquid nitrogen for storage at −80°C until processing. These were thawed by the addition of three volumes of KCl buffer [1.15% (w/v) potassium chloride, 10 mM potassium phosphate, pH 7.4] and homogenized by a rotor-stator. Debris was pelleted by centrifugation (11,000*g* at 4°C for 15 minutes) and the supernatant was withdrawn for ultracentrifugation (100,000*g* at 4°C for 60 minutes). After ultracentrifugation, the pellet (microsomal fraction) was resuspended in KCl buffer containing 0.25 M sucrose. Protein content was quantified by the Bradford assay (Bio-Rad, Hemel Hempstead, UK).

#### In Vitro Studies.

All in vitro analyses were carried out in 100 mM potassium phosphate buffer, pH 7.4, containing 3.3 mM MgCl_2_, with agitation at 400 rpm at 37°C on a thermoshaker. All samples were handled in amber tubes under conditions of subdued light for the duration of the procedure. Incubations were initiated by the addition of NADPH to a final concentration of 1 mM and were terminated by transferring an aliquot of the reaction mixture, typically 50 *µ*l, to two volumes of ice-cold acetonitrile containing internal standard (tamoxifen-*d*_5_ at 0.2 *µ*g/ml), followed by vortexing (5 seconds) and incubation on ice. Assays to determine the apparent kinetic parameters of endoxifen formation in hCYP2D6 mouse liver microsomes (MLMs) were performed in triplicate under conditions of linearity for time (6 minutes) and hCYP2D6 protein (0.0625 mg/ml). All subsequent assays were carried out under the same conditions, irrespective of the microsome source, with 5 *µ*M *N*-desmethyltamoxifen (NDT) as substrate. For AD inhibition assays, fluoxetine, amitriptyline, clomipramine, and imipramine were coincubated with substrate. Paroxetine (at 2× concentration) was preincubated with MLMs (0.125 mg/ml) and NADPH (1 mM) for 20 minutes before addition of an equal volume of buffer containing NDT (10 *µ*M) and fresh NADPH (2 mM); therefore, concentrations of all components in the final reaction mixture were the same for all ADs. Solvent (methanol) concentrations were 0.2% or lower in all incubations.

#### In Vivo Studies.

All animal work was carried out on 8- to 12-week-old female mice. Paroxetine maleate salt was dissolved to 2.16 mg/ml in phosphate-buffered saline to give a solution of 1.6 mg/kg paroxetine, which was administered at a dose of 8 mg/kg orally (5 *µ*l/g body weight). NDT was suspended in corn oil at 2 mg/ml for oral administration at 10 mg/kg (5 *µ*l/g body weight). For sample collection for pharmacokinetic (PK) analysis, 10 *µ*l whole blood was withdrawn from the tail vein at the indicated time points. Samples were immediately added to a tube containing heparin solution (10 *µ*l, 15 IU/ml) and stored at −20°C until processing.

#### Sample Processing for Liquid Chromatography–Tandem Mass Spectrometry.

In vivo PK samples were thawed by the addition of 70 *µ*l acetonitrile containing the internal standard (tamoxifen-*d*_5_ at 0.2 *µ*g/ml). After incubation on ice for 10 minutes, in vivo and in vitro samples were vortexed (5 seconds) and centrifuged for 10 minutes at 16,000*g*. The supernatant was added to 96-well plates for liquid chromatography (LC)–tandem mass spectrometry (MS/MS). As with microsomal incubations, all samples were handled in amber tubes under conditions of subdued light throughout the procedure.

#### LC-MS/MS.

Analysis of in vitro incubation and in vivo blood PK samples was carried out on a Waters Acquity ultra-performance LC system and a Micromass Quattro Premier mass spectrometer (both Micromass, Manchester, UK). LC separation was performed on a Kinetex 1.7 *μ*M C19 100A column (50 × 2.1 mm; Phenomenex, Macclesfield, UK) at a temperature of 45°C with an injection volume of 5 *µ*l and flow rate of 0.5 ml/min. Mobile phases were water containing 0.1% (v/v) formic acid (A) and acetonitrile containing 0.1% (v/v) formic acid (B). Gradient elution was carried out from 70%/30% A/B to 30%/70% A/B over 2 minutes. Multiple reaction monitoring data in electrospray ionization–positive mode were acquired for NDT [358.20 > 57.98; cone voltage (CV), 40 V; and collision energy (CE), 25 kV], endoxifen [374.22 > 58.04; CV, 42 V; and CE, 23 kV], and tamoxifen-*d*_5_ [377.22 > 71.87; CV, 45 V; and CE, 26 kV]. Acquired data were analyzed in QuanLynx (Waters, Milford, MA) relative to analyte standard curves spanning the range of concentrations under study. Analyte recovery was high (approximately 90%) and the LC-MS/MS assay was highly reproducible between runs, as reflected in the continuity of signal from the calibration standards.

#### Data Analysis.

In vitro kinetic data exhibited a substrate inhibition profile and therefore were fitted with the following equation using GraFit software (version 7; Erithacus Software, Horley, UK): *V* = *V*_max_ × [S]/[*K*_S1_ + [S] × (1 + [S]/*K*_S2_)], where [S] is the substrate concentration, *K*_S1_ is the dissociation constant for productive enzyme (substrate complex), and *K*_S2_ is the dissociation constant for substrate bound to the inhibitory site. Spearman’s rank correlations and inhibition parameters of ADs were calculated using GraphPad Prism software (version 6; GraphPad Inc., La Jolla, CA). PK parameters of in vivo data were calculated with a simple noncompartmental model using PK functions in Microsoft Excel (Microsoft, Redmond, WA) and *P* values were calculated using an unpaired, one-tailed *t* test.

## Results

### 

#### NDT Is Converted to Endoxifen by CYP2D6 in hCYP2D6 MLMs In Vitro.

**Fig. 2.**
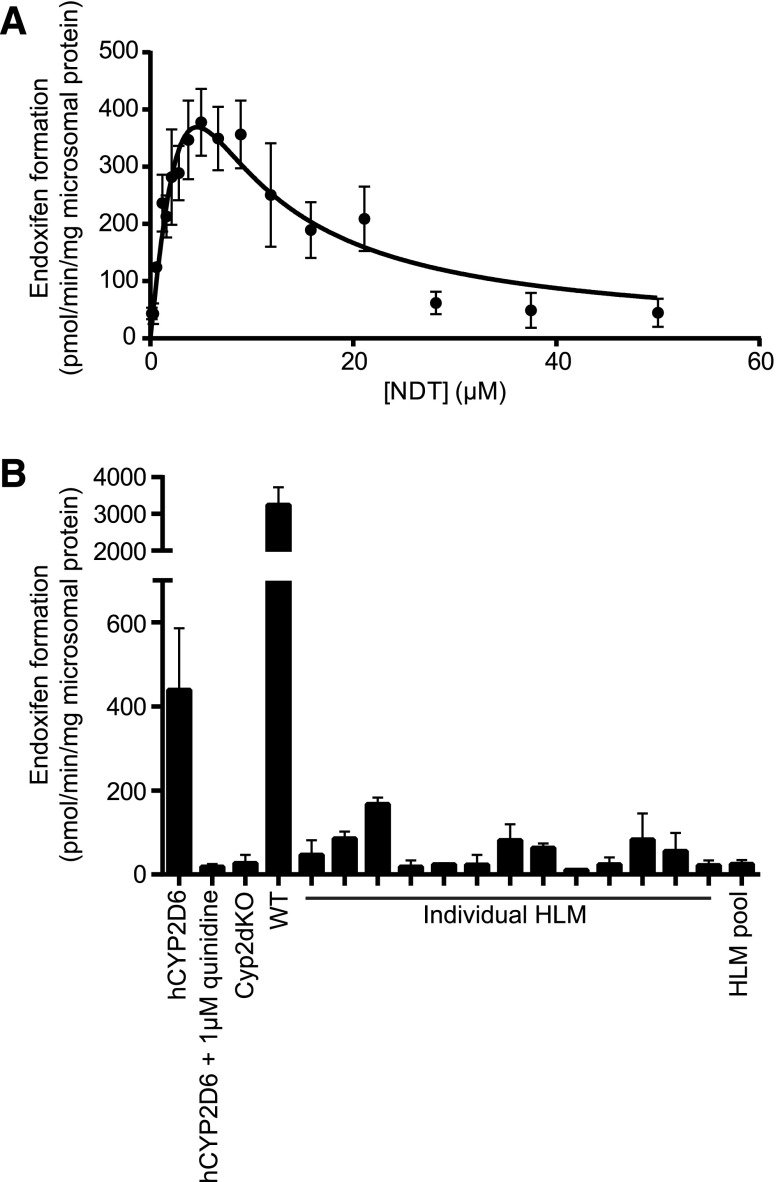
CYP2D6 converts NDT to endoxifen in hCYP2D6 mice. (A) Kinetic analysis of endoxifen formation in hCYP2D6 MLMs (pooled from four animals). Data represent combined means ± S.D. of 10 technical replicates carried out on four separate occasions. (B) Endoxifen formation in MLMs and HLMs. MLMs were pooled from three (Cyp2dKO, WT) or four (hCYP2D6) individual animals. HLMs were individual (13 donors) or pooled (150 donors). Data represent means ± S.D. of duplicate incubations and are representative of an experiment carried out on two separate occasions. WT, wild type.

Under conditions of linearity for time and protein, formation of endoxifen from NDT in hCYP2D6 liver microsomes exhibited a kinetic profile suggestive of substrate inhibition (Fig. 2A). Apparent kinetic parameters were obtained, with a *K*_S1_ of 5.1 ± 0.4 *µ*M, *K*_S2_ of 3.9 ± 0.3 *µ*M, and *V*_max_ of 1128 ± 57 pmol/min per mg. Under the same incubation conditions, and with 5 *µ*M NDT as substrate, coincubation with 1 *µ*M quinidine reduced endoxifen formation by > 95% (Fig. 2B). Liver microsomes from Cyp2dKO mice did not produce detectable levels of endoxifen, whereas liver microsomes from wild-type mice produced 7.4-fold more endoxifen than hCYP2D6 liver microsomes. In incubations with a high/low P450 activity HLM panel, hydroxylation of NDT correlated most strongly with that of bufuralol, the probe drug for CYP2D6, although a statistically significant correlation was also observed with the probe for CYP2B6 (Table 1; Supplemental Fig. 1).

**TABLE 1:** Spearman’s rank correlation of conversion of NDT to endoxifen with P450 probe substrate metabolism in a panel of 13 HLMs Spearman’s rank correlation coefficient of NDT hydroxylation with metabolism of P450 probe substrates is shown. Probe substrate values were provided by the vendor.

Enzyme	Probe Substrate Activity	SRCC with NDT Hydroxylation	*P* Value
1A2	Phenacetin *O*-deethylation	−0.289	0.360
2A6	Coumarin 7-hydroxylation	0.161	0.600
2B6	*S*-mephenytoin *N*-demethylation	0.572	0.045
2C8	Paclitaxel 6*α*-hydroxylation	0.380	0.186
2C9	Diclofenac 4′-hydroxylation	0.526	0.076
2C19	*S*-mephenytoin 4′-hydroxylation	−0.220	0.444
2D6	Bufuralol 1′-hydroxylation	0.698	0.010
2E1	Chlorzoxazone 6-hydroxylation	0.311	0.281
3A4	Testosterone 6*β*-hydroxylation	−0.005	0.993
4A11	Lauric acid 12-hydroxylation	−0.290	0.316

SRCC, Spearman’s rank correlation coefficient.

#### ADs Inhibit the Conversion of NDT to Endoxifen by hCYP2D6 Liver Microsomes.

**Fig. 3.**
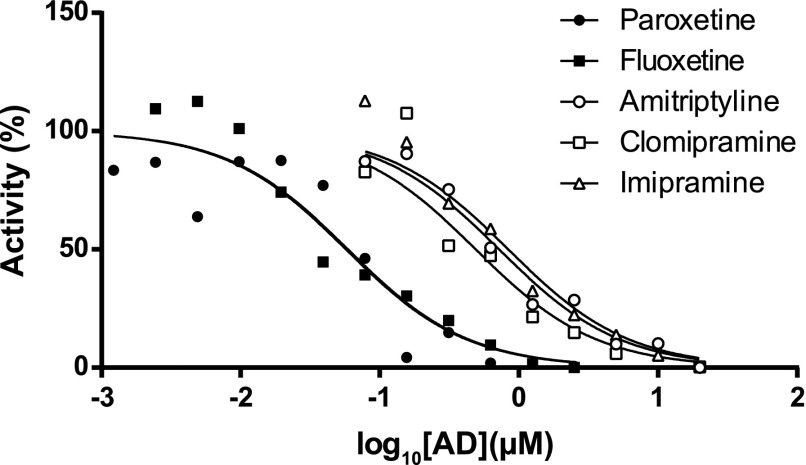
Conversion of NDT to endoxifen in hCYP2D6 MLMs is inhibited by ADs. Paroxetine was preincubated with MLMs before addition of NDT, as described in the *Materials and Methods*. All other compounds were coincubated with NDT. Data represent means of duplicate incubations and are representative of an experiment carried out on two separate occasions.

To determine whether ADs could inhibit the formation of endoxifen from NDT in hCYP2D6 liver microsomes, two SSRIs (paroxetine and fluoxetine) and three tricyclic ADs (amitriptyline, clomipramine, and imipramine) were individually titrated into the optimized reaction mixture. Because paroxetine is a mechanism-based inhibitor of CYP2D6 (Bertelsen et al., 2003), a 20-minute preincubation step was carried out for this compound as described in the *Materials and Methods*. All compounds inhibited the reaction, with *K*_i_ values (95% confidence intervals) of 57 nM (36.9–88.2 nM) for paroxetine, 59 nM (39–89 nM) for fluoxetine, 720 nM (515–1008 nM) for amitriptyline, 489 nM (351–680 nM) for clomipramine, and 838 nM (576–1218 nM) for imipramine (Fig. 3).

#### Paroxetine Inhibits the CYP2D6-Mediated Conversion of NDT to Endoxifen in hCYP2D6 Mice In Vivo.

**Fig. 4.**
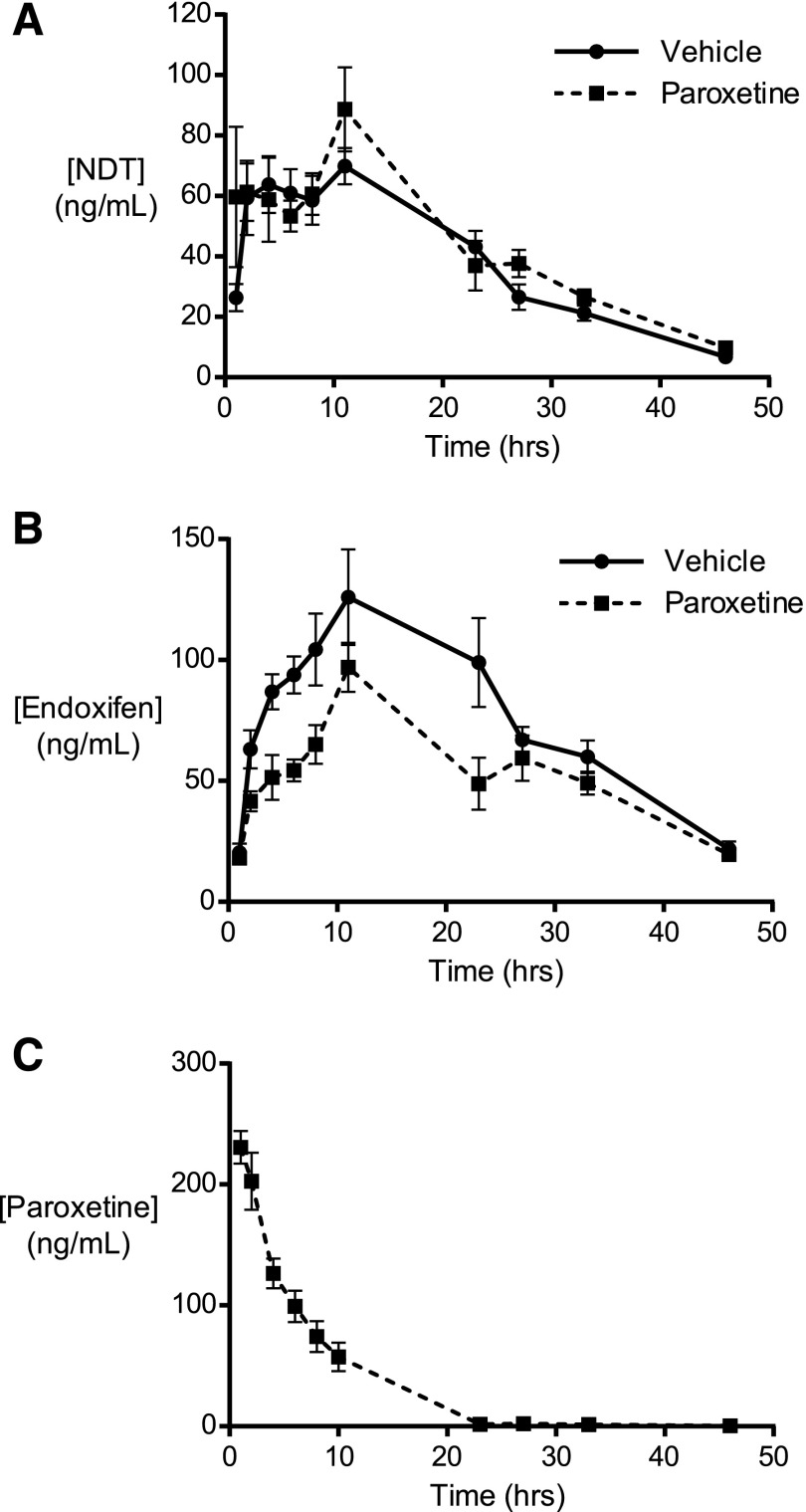
Paroxetine inhibits conversion of NDT (A) to endoxifen (B) in hCYP2D6 in vivo. (C) Animals were dosed with paroxetine (8 mg/kg, *n* = 6) or vehicle (*n* = 6) and then, 1 hour subsequently, all were dosed with NDT (10 mg/kg). Data shown are means ± S.E.M.

In patients receiving the most common clinical dose of 20 mg tamoxifen per day, the average blood plasma steady-state *C*_max_ of NDT is approximately 200 ng/ml (Lien et al., 2013; Jager et al., 2014). Preliminary studies in our laboratory (data not shown) indicated that single doses of NDT required to achieve similar concentrations were not well tolerated by all animals. Hence, a dose of 10 mg/kg NDT was used here to give a reasonable level of exposure, at approximately half of steady-state human levels, while avoiding toxic effects. For paroxetine, although steady-state *C*_max_ is not dose proportional (Sindrup et al., 1992), it is approximately 150 ng/ml in patients receiving the relatively high dose of 40 mg/d (http://www.gsk-clinicalstudyregister.com/study/29060/474?study_ids=29060/474#rs). We found that a single dose of 8 mg/kg paroxetine was appropriate to achieve maximum plasma concentrations in hCYP2D6 mice that were similar to this reported human value. Therefore, to establish whether the in vitro interaction of NDT with paroxetine occurred in hCYP2D6 mice in vivo, six animals were administered paroxetine and six were administered phosphate-buffered saline vehicle alone, 1 hour prior to all 12 receiving NDT. Plasma levels of both of these compounds, and of endoxifen, were monitored over the following 48-hour period. There were no observed differences in the PK profile or apparent parameters of NDT between groups (Fig. 4A). However, pretreatment with paroxetine resulted in decreased exposure to endoxifen, with a highly significant reduction in the area under the curve (AUC_all_) and a moderate but nonsignificant decrease in *C*_max_ (Fig. 4B; Table 2). Monitoring of paroxetine levels in the pretreated group confirmed that the intended level of exposure had been reached (Fig. 4C).

**TABLE 2:** PK parameters of endoxifen in hCYP2D6 mice Parameters (means ± S.D.) are shown for vehicle (*n* = 6) and paroxetine (*n* = 6) pretreated groups.

Group	*t*_1/2_	*C*_max_	AUC_all_
	*h*	*ng/ml*	*h⋅ng/ml*
Vehicle	14.3 ± 4.7	132 ± 46	3508 ± 797
Paroxetine	13.8 ± 3.1	104 ± 12	2525 ± 321*
*P* value	0.416	0.094	0.009

*t*_1/2_, half-life. *P <0.05.

## Discussion

Tamoxifen has been in clinical use for the treatment of cancer since the 1970s but the relatively recent discovery of endoxifen (Stearns et al., 2003), coupled with an increased understanding of the phenotypic variability of CYP2D6 (Zanger et al., 2004), has suggested opportunities for further optimization of therapy. There is a general consensus that the interaction of tamoxifen with CYP2D6 and strong inhibitors thereof may have some bearing on the outcome of therapy, but prospective trials are needed to determine the true extent of these effects, whether genotype-guided therapy should be adopted, and whether coadministration of certain ADs with tamoxifen should be avoided (Kelly et al., 2010; Province et al., 2014).

Reported steady-state concentrations of tamoxifen metabolites in patient serum are highly variable but metabolism through NDT is defined as the major route (with mean values of approximately 200, 10–50, and 2–5 ng/ml for NDT, endoxifen, and 4-HT, respectively) (Fig. 1) (Stearns et al., 2003; Madlensky et al., 2011; Lien et al., 2013; Jager et al., 2014). However, in preliminary experiments with a single 15-mg/kg oral dose of tamoxifen in hCYP2D6 mice, we observed a 2.7-fold higher value for blood concentrations of 4-HT than for NDT (*C*_max_ values for 4-HT and NDT were 235 and 86 ng/ml, respectively; data not shown), indicating that the major and minor pathways were reversed in this species. Hence, we deemed it necessary to bypass this conflict in primary metabolism by using NDT as the substrate in our experiments. Recently, this same problem has been encountered in a mouse model containing humanizations for pregnane X receptor (PXR), constitutive androstane receptor (CAR), CYP3A4/3A7, and CYP2D6 (hPXR/hCAR/h3A4/3A7/2D6) (Chang et al., 2016). After administration of a single dose of 20 mg/kg tamoxifen to this complex model, 4-HT levels were found to be 5.4-fold higher than those of NDT (*C*_max_ values for 4-HT and NDT were 248 and 46 ng/ml, respectively). As far as we are aware, the mouse enzymes that mediate this preferential hydroxylation of tamoxifen are yet to be identified.

There are over 100 identified allelic variants of *CYP2D6* (http://www.cypalleles.ki.se/cyp2d6.htm), with varying levels of activity in the metabolism of compounds of both endogenous and exogenous origin (Yu et al., 2004; Zanger et al., 2004). Depending on patients’ CYP2D6 status, they may be classed as poor, intermediate, extensive, or ultrarapid metabolizers (Cascorbi, 2003; De Gregori et al., 2010). This phenotypic status can be determined through the analysis of the urinary ratio of debrisoquine to its CYP2D6-generated metabolite, 4-hydroxydebrisoquine (Dalén et al., 1999). In a previous study, we found that wild-type mice and Cyp2dKO mice were representative of human poor metabolizers in this regard, whereas hCYP2D6 mice were representative of extensive metabolizers (Scheer et al., 2012). In this study, we show that, in vitro, liver microsomes from Cyp2dKO mice are incapable of generating endoxifen from NDT, whereas wild-type MLMs do so at a rate that greatly exceeds that of HLMs. This comparatively high activity of murine P450s has been seen for other drugs and maybe due, at least in part, to the multiplicity of these enzymes (Hrycay and Bandiera, 2009; Hasegawa et al., 2011; Scheer et al., 2012). Humanization for CYP2D6 both removes this high-activity murine component and incorporates the functional human component, rendering the amount of endoxifen produced more in line with that of the members of the HLM panel that possess the highest level of activity toward the CYP2D6 probe (bufuralol). As with the urinary ratio of debrisoquine to 4-hydroxydebrisoquine, therefore, it appears that Cyp2dKO and hCYP2D6 mice are at either end of the CYP2D6 phenotypic spectrum in relation to NDT metabolism in vitro. Our data for hCYP2D6 MLMs yielded a *K*_S1_ value of 5.1 ± 0.4 *µ*M, which is consistent with the *K*_m_ values of 4.5 and 5.9 *µ*M reported by Desta et al. (2004) in their analyses of two individual HLM preparations. These authors also noted that, in a third HLM preparation, endoxifen was formed at a very slow rate. Our observations are also consistent with those of Chang et al. (2016), who reported a *K*_m_ value of 5.9 *µ*M for 4-HT hydroxylation in their recent work with liver microsomes from the hPXR/hCAR/h3A4/3A7/2D6 model. Furthermore, we found that NDT hydroxylation correlated most strongly with bufuralol hydroxylation in a panel of HLMs, confirming the wider importance of CYP2D6 in this context. We also observed, however, a correlation with *S*-mephenytoin *N*-demethylation, the probe activity for CYP2B6. Although this correlation barely achieved statistical significance, this raises the possibility that there may be a role for this enzyme in the generation of endoxifen from NDT, which deserves further investigation.

Here, the SSRIs paroxetine and fluoxetine inhibited the CYP2D6-dependent formation of endoxifen in hCYP2D6 MLM incubations, with *K*_i_ values of 57 nM and 59 nM, respectively. These values are somewhat lower than the 360 nM (paroxetine) and 240 nM (fluoxetine) previously reported by Bertelsen et al. (2003) in the inhibition of dextromethorphan *O*-demethylation by HLMs but, in the case of paroxetine, other reported values are closer to our observed value (Crewe et al., 1992; Fogelman et al., 1999). Tricyclic ADs were an order of magnitude less effective in inhibiting NDT hydroxylation. Paroxetine is a strong inhibitor of CYP2D6 (http://www.fda.gov/Drugs/DevelopmentApprovalProcess/DevelopmentResources/DrugInteractionsLabeling/ucm093664.htm#cypEnzymes) and is the most likely AD to exert a negative influence on the outcome of tamoxifen therapy (Borges et al., 2006; Kelly et al., 2010). Here, we observed a substantial decrease in exposure (AUC_all_) to endoxifen in hCYP2D6 mice treated with NDT and paroxetine, relative to the control, demonstrating that this drug–drug interaction occurs in vivo. Crucially, the maximum plasma concentrations of NDT and paroxetine were similar to those evident in patients at steady state (Lien et al., 2013; Jager et al., 2014; http://www.gsk-clinicalstudyregister.com/study/29060/474?study_ids=29060/474#rs). We did not observe the converse effect with NDT (i.e., an increased exposure in the presence of paroxetine). This finding suggests that the routes of elimination for NDT and endoxifen may be different and is in agreement with data from the hPXR/hCAR/h3A4/3A7/2D6 model (Chang et al., 2016). Indeed, 4-OH metabolites of tamoxifen, including endoxifen, are known to be glucuronidated and sulfated (Poon et al., 1993; Kisanga et al., 2005). It would therefore be informative to determine, in future experiments, whether the profiles of these and other circulating and excreted conjugates are altered with paroxetine coadministration. Furthermore, it would be interesting to ascertain whether paroxetine has a more profound effect on endoxifen levels in hCYP2D6 mice at steady state. As discussed above, hCYP2D6 mice align with the CYP2D6 extensive-metabolizer phenotype, yet we saw decreases of only 21% in *C*_max_ and only 28% in AUC_all_. Although this has to be viewed in consideration of the rapid elimination of paroxetine in our study, these values may be more in line with the 64%–71% decrease seen in patients at steady-state levels of both NDT and paroxetine (Stearns et al., 2003; Borges et al., 2006). Although plasma levels of paroxetine in hCYP2D6 mice were an order of magnitude higher than the observed in vitro *K*_i_ for inhibition of NDT hydroxylation (670 nM versus 57 nM), significant quantities of endoxifen were still produced. One possible explanation for this is the high level of binding of paroxetine with murine plasma proteins, which (at >96%) is similar to the reported human value, because inhibition of CYP2D6 may be dependent on the free drug concentration (van Harten, 1993; Qin et al., 2016). Consistent with the in vitro analyses that indicated that hCYP2D6 liver microsomes were at least 2.6-fold more efficient than any member of the HLM panel in generating endoxifen, it should be noted that endoxifen levels were higher (between 2.5- and 13-fold) than in human subjects (Jin et al., 2005; Lien et al., 2013; Jager et al., 2014).

In the in vivo study presented here, we attempted to incorporate a pharmacodynamic endpoint by Western blotting for ER targets (Cdc2, Mad2, and p21) in the endometrium, where tamoxifen is known to exert proestrogenic effects, but no changes were observed (data not shown). This may be because of the short-term nature of the study: chronic administration of tamoxifen/NDT may be required to generate the precancerous changes observed in human patients. We also investigated the utility of the C57BL/6-derived E0771 cell line for potential syngeneic tumor studies. As observed by Gu et al. (2009), this cell line expresses ER*α* at a level far lower than in the estrogen-dependent MCF7 human cell line. Indeed, we found that E0771 cells exhibited no dependence whatsoever on estradiol for their growth in vitro (Supplemental Fig. 2). Future work incorporating pharmacodynamic endpoints, such as the antitumor activity of NDT in xenografted immunodeficient hCYP2D6 and Cyp2dKO mice, would allow the further evaluation of tamoxifen interactions with *CYP2D6* phenotype and ADs.

In summary, we have shown that humanization for CYP2D6 is both necessary and sufficient to render the mouse human-like in its disposition to NDT. In modeling human-specific aspects of tamoxifen metabolism in vivo, we have demonstrated that ADs, particularly those of the SSRI class, have the capacity to alter systemic exposure to pharmacologically potent metabolites, which may influence therapeutic outcome. Our work exemplifies the utility of humanized mouse models for the nonclinical study of drug metabolism.

## Abbreviations

4-HT: 4-hydroxytamoxifen
AD: antidepressant
AUC: area under the curve
CAR: constitutive androstane receptor
CE: collision energy
CV: cone voltage
ER: estrogen receptor
HLM: human liver microsome
LC: liquid chromatography
MLM: mouse liver microsome
MS/MS: tandem mass spectrometry
NDT: *N*-desmethyltamoxifen
P450: cytochrome P450
PK: pharmacokinetic
PXR: pregnane X receptor
SSRI: selective serotonin reuptake inhibitor
